# Results of a Double-Blind, Randomized, Placebo-Controlled Phase 1 Study to Evaluate the Safety and Pharmacokinetics of Anti-Zika Virus Immunoglobulin

**DOI:** 10.4269/ajtmh.20-1578

**Published:** 2021-10-04

**Authors:** Jane White, Priya Tunga, Deborah M. Anderson, Ken Iledan, Tobi Loreth, Geraldine S. Parrera, Hugo Astacio, Bojan Drobic, Jason S. Richardson

**Affiliations:** ^1^ Emergent BioSolutions Inc., Gaithersburg, Maryland;; ^2^ Emergent BioSolutions Canada Inc., Winnipeg, Manitoba, Canada

## Abstract

Zika virus (ZIKV) is transmitted primarily through infected *Aedes aegypti* or *Aedes albopictus* mosquitoes. ZIKV infection during pregnancy was linked to adverse fetal/infant outcomes, including microcephaly, brain anomalies, ocular disorders, intrauterine growth restriction, and other congenital malformations. Human anti-Zika virus immunoglobulin (ZIKV-Ig) is being developed for prophylaxis of ZIKV in at-risk populations, including women of childbearing potential and pregnant women. A phase 1 single-center, double-blind, randomized, placebo-controlled study was conducted to assess the safety and pharmacokinetics (PK) of a single 50.0-mL ZIKV-Ig intravenous dose in healthy adult male or non-pregnant female subjects 18 to 55 years of age. Subjects received either ZIKV-Ig (*n* = 19) or saline placebo (*n* = 11). Safety was evaluated based on adverse events (AEs), laboratory test results, physical examinations, and vital signs. Overall, there were 11 subjects (36.7%) with treatment-related AEs including eight subjects (42.1%) in the ZIKV-Ig group and three subjects (27.3%) in the placebo group. Of the AEs considered treatment related, three subjects (15.8%) experienced headache (mild). There were no serious AEs, no deaths, and no discontinuations resulting from AEs. Overall, the safety profile of ZIKV-Ig in this study population of healthy adult subjects appeared to be safe and well tolerated. The results of the pharmacokinetic analysis determined that ZIKV-Ig had a maximum observed concentration of 182.3 U/mL (coefficient of variation, 21.3%), the time at which C_max_ occurred of 2.3 hours ± 1.0 (SD), an area under the concentration–time curve_0–∞_ of 77,224 h × U/mL (coefficient of variation, 17.9%), and a half-life of 28.1 days, which is similar to other human-derived commercial Ig intravenous products.

## INTRODUCTION

Zika virus (ZIKV) is an RNA *Flavivirus* related closely to Dengue virus (DENV), Yellow fever virus, Japanese encephalitis virus, and West Nile virus (WNV). ZIKV is transmitted primarily through the bite of an infected *Aedes* sp. mosquito (*Ae. aegypti* or *Ae. albopictus*); however, sexual transmission has also been frequently reported.^1^^,^^2^ Zika fever (also known as ZIKV disease) is an illness caused by this virus. Currently there are no licensed products for the prevention or treatment of ZIKV infection.

The first recorded outbreak of ZIKV disease was reported from the Island of Yap (Federated States of Micronesia) in 2007, followed by a large outbreak in French Polynesia in 2013, and other countries and territories in the Pacific.^3^ In 2015, Brazil reported a large outbreak, and viral transmission soon appeared throughout the Americas and other regions of the world.^3^ In the United States these outbreaks resulted in limited local transmission in Florida and Texas, including widespread transmission in Puerto Rico and the U.S. Virgin Islands, and an increase in travel-associated cases.^3^ According to the WHO, as of July 2019, 87 countries and territories have evidence of mosquito-borne transmission of ZIKV, distributed across four of the six WHO-defined regions, including the African Region, Region of the Americas, Southeast Asia Region, and Western Pacific Region.^4^^,^^5^ All areas with previous reports of ZIKV transmission have the potential for re-emergence. There is also the potential risk for ZIKV to spread to additional countries, as 61 countries and/or territories globally have evidence of established competent mosquito vectors but have not yet documented ZIKV transmission to humans.^4^^,^^5^ It is also possible that some of these countries have or have had transmission that has not yet been detected or reported.^5^

Most individuals (∼80%) infected by ZIKV, are asymptomatic, whereas symptomatic individuals typically present with acute onset of fever, maculopapular rash, joint pain, headache, or non-purulent conjunctivitis that usually lasts from several days to a week.^2^ However, ZIKV infection has been linked to more severe disease outcomes such as Guillain-Barré syndrome^6^^,^^7^ and other neurological impairments,^8^ albeit infrequently. In addition, ZIKV infection during pregnancy has been linked to adverse fetal/infant outcomes, including microcephaly,^9^^,^^10^ serious brain anomalies,^11^^,^^12^ ocular disorders,^12^ intrauterine growth restriction, and other congenital malformations resulting in congenital Zika syndrome.^13^^,^^14^ The percentage of fetuses/infants with possible ZIKV-associated birth defects ranges from 4% to 8%, depending on maternal time of ZIKV exposure during pregnancy.^15^ The ability of ZIKV to infect and damage developing fetuses implies the virus can cross and/or bypass the placental barrier, but the mechanism remains unclear.^16^ Other flaviviruses, including DENV, are not associated with vertical transmission or congenital disorders, which suggests this mechanism may be specific to ZIKV.^17^

Emergent BioSolutions Canada Inc. (EBCI) developed human anti-Zika virus immunoglobulin (ZIKV-Ig), a human hyperimmune product of purified gamma IgG fraction of human plasma containing polyclonal antibodies reactive to ZIKV. ZIKV-Ig is prepared from pooled plasma collected at U.S. Food and Drug Administration (FDA)–licensed plasma collection centers from healthy adult donors who have elevated levels of antibodies reactive to ZIKV, and was manufactured using EBCI’s hyperimmune manufacturing platform process that is used to manufacture FDA-licensed products that include CNJ-016^TM^ (Vaccinia Immune Globulin Intravenous (Human) [VIGIV]); Emergent BioSolutions, Winnipeg, Canada), ANTHRASIL^®^ (Anthrax Immune Globulin Intravenous [Human]; Emergent BioSolutions, Winnipeg, Canada), WinRho^®^ SDF (Rho(D) Immune Globulin Intravenous [Human]), Saol Therapeutics Inc., Roswell, GA, USA), HepaGam B^®^ (Hepatitis B Immune Globulin Intravenous [Human]), Emergent BioSolutions, Winnipeg, Canada), and VariZIG^®^ (Varicella Zoster Immune Globulin [Human)]; Emergent BioSolutions, Winnipeg, Canada).

In this first-in-human phase 1 clinical study (ZK-001; ClinicalTrials.gov identifier NCT03624946), the safety, tolerability, and pharmacokinetics (PK) of a 50.0-mL intravenous (IV) dose (∼50–100 mg/kg) of ZIKV-Ig was assessed in healthy adults.

## MATERIALS AND METHODS

### Study design and participants.

Ours was a randomized, double-blind, placebo-controlled single-dose study conducted at a phase 1 unit (Syneos Health) in Toronto, Ontario, Canada. The study protocol was approved by Advarra Institutional Review Board and was conducted in accordance with the International Council for Harmonisation good clinical practice guidelines and all applicable local regulatory requirements and laws. Participants provided written informed consent before any study procedures occurred and were re-consented, as necessary in the occurrence of a study protocol amendment.

Briefly, notable inclusion criteria included enrollment of healthy adult male or non-pregnant female volunteers 18 to 55 years of age who had a body mass index of 18.0 to 30.0 kg/m^2^, and were in good health as determined by no clinically significant findings from medical history, physical examination, electrocardiogram, clinical laboratory assessments, and vital sign measurements.

Participants were excluded based on screening assessments if they were administered (or planned to use during the study) an investigational product within 30 days of screening, blood products within the 12 months up to screening, and/or live vaccines within 28 days prior to screening. Participants were excluded if they had a history of hypersensitivity to blood (or plasma) products, IgA deficiency, hypercoagulable conditions, myocardial infarction, stroke, renal impairment/failure, a chronic or acute severe neurological condition, and/or the opinion of the investigator that it would be unwise to allow participation of the individual in the study.

Criteria were included to ensure subjects did not have preexisting antibodies to flaviviruses to mitigate interference with the PK assessment, such as excluding individuals with a history of *Flavivirus* infection or previous vaccination with licensed or investigational *Flavivirus* vaccine. At the screening visit, a serum sample was taken to perform viral marker serology testing for ZIKV IgG and IgM antibodies, DENV IgM and IgG antibodies, and WNV IgM and IgG antibodies. In addition, a ZIKV nucleic acid test (NAT) was performed on serum and urine samples. At baseline, viral marker serology testing for ZIKV IgG, ZIKV IgM, and viral marker ZIKV NAT of serum and urine was also performed. Screening serology results for DENV and WNV were reported along with baseline ZIKV NAT results for serum and urine, and baseline ZIKV IgM and IgG serology results.

Because of the limited number of plasma donors with elevated levels of antibodies to ZIKV, donor plasma with high isoagglutinin titers (e.g., anti-A and anti-B antibodies) were not excluded from the plasma pool used for the manufacture of the ZIKV-Ig clinical lot. Therefore, for this study, ZIKV-Ig was intended for IV administration only to subjects with blood types O+ or O– individuals to mitigate the occurrence of hemolytic reactions in subjects. For this reason, participants were excluded if they had blood types A, B, or AB. The full inclusion and exclusion criteria for this study is provided in Supplemental Appendix S1.

### Randomization and masking/blinding.

A randomization scheme was provided by an unblinded contract research organization (CRO) statistician who was not involved in study treatment administration or subject assessments. The randomization program was written and documented per the CRO’s standard operating procedures and in accordance with EBCI’s Development of Statistical Analysis System Programs and Good Statistical Analysis System Programming Practice standard operating procedures. Subjects were assigned randomly to receive a single dose of ZIKV-Ig or a single dose of placebo (normal saline, 0.9% sodium chloride) by IV infusion.

To maintain the blind, all study drugs (ZIKV-Ig or placebo) were dispensed in a comparable manner. The pharmacy staff assigned to the study at the CRO’s phase 1 clinical site were unblinded to access the randomization assignment and prepare the study treatment. Both the site principal investigator (PI) and subjects were masked to the treatment assignment. Staff at EBCI were also masked to study drug assignment throughout the study to facilitate ongoing monitoring of the safety of the subjects.

### Investigational product dose.

The clinical dose of ZIKV-Ig was 50.0 mL (undiluted) containing a total of 4.65 g IgG protein (93 mg/mL). The liquid product contained 30 to 130 mg/mL protein (of which, at least 96% is purified human IgG) stabilized with 10% maltose and 0.03% polysorbate 80. The potency of the drug product, determined by a cell-based ZIKV microneutralization assay, was 10,612 U/mL. The per-subject duration of infusion was expected to be complete within 33 minutes based on a per-protocol rate of infusion (1.0 mL/min for the first 15 minutes with an increase to the rate to 2.0 mL/min for the remainder of the IV infusion).

### Staggered dosing schedule and safety monitoring.

**Figure 1.**
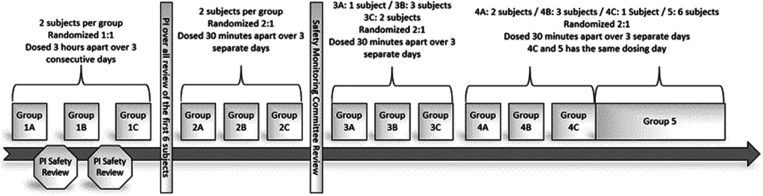
Illustrative representation of the ZK-001 treatment schedule. Group 1A (*n* = 2), group 1B (*n* = 2), group and 1C (*n* = 2) were randomized 1:1 in a double-blind fashion to receive either human anti-Zika virus immunoglobulin (ZIKV-Ig) or placebo. Two subjects per day were dosed at least 3 hours apart and each subgroup was dosed at least 1 day apart over 3 days, with the principal investigator (PI) safety data review occurring between subgroups followed by an overall safety data review of all 6 group 1 subjects prior to group 2 dosing. Groups 2A (*n* = 2), 2B (*n* = 2), and 2C (*n* = 2) were randomized 2:1 in a double-blind fashion to receive either ZIKV-Ig or placebo and were dosed 30 minutes apart on three separate days. An independent Safety Monitoring Committee reviewed safety data for the first 12 dosed subjects (groups A and B) prior to group 3 dosing. Groups 3A (*n* = 1), 3B (*n* = 3), and 3C (*n* = 2) were randomized 2:1 in a double-blind fashion to receive either ZIKV-Ig or placebo and were dosed 30 minutes apart on three separate days. Groups 4A (*n* = 2), 4B (*n* = 3), and 4C (*n* = 1) were randomized 2:1 in a double-blind fashion to receive either ZIKV-Ig or placebo and were dosed 30 minutes apart on three separate days. Group 5 (*n* = 6) was randomized 2:1 in a double-blind fashion to receive either ZIKV-Ig or placebo and was dosed 30 minutes apart on a single day (group 4C).

The first six subjects were randomized 1:1 in pairs in a double-blind fashion to receive either ZIKV-Ig or placebo control. Dosing was staggered over 3 days, during which two subjects per day were randomized 1:1 and dosed to receive either ZIKV-Ig or placebo 3 hours apart (Figure 1). Two subjects per day were dosed at least 3 hours apart, and each subgroup (dosed at least 1 day apart) was staggered over 3 days for subgroups 1A, 1B, and 1C. The PI reviewed the available safety information, including reported adverse events (AEs) within 3 hours after study treatment administration, concomitant medications, and vital signs (15 minutes into the IV infusion, at the end of the IV infusion, and 1 hour and 3 hours after study drug administration) between group 1 subgroups. The PI assessed the data for safety concerns after all six subjects from group 1 collectively, prior to randomization of group 2. Group 2 consisted of six subjects randomized 2:1 in a double-blind fashion to receive either ZIKV-Ig or placebo, dosed at least 30 minutes apart. An independent Safety Monitoring Committee review consisted of the PI and EBCI’s blinded medical monitor (MM). The Safety Monitoring Committee reviewed safety data for the first 12 dosed subjects (collected up to 72 hours post-study drug administration), prior to the remaining 18 subjects being randomized 2:1 and administered the study drug (at least 30 minutes apart) in three separate groups of six subjects (groups 3, 4, and 5).

### Procedures and assessments.

The schedule of events is provided in Table 1, which displays the planned clinic visits and the per-visit assessments. Briefly, this study consisted of a screening visit to establish subject eligibility (Supplemental Appendix S1) within 35 days prior to study drug administration on day 1. Subjects arrived at the phase 1 clinic the day before (day –1) drug administration on day 1 and stayed until 24 hours after drug administration (day 2). Study assessments for safety and PK analysis were performed at the time of admission to the clinic on Day –1, on Day 1, pre-dose, and post-dose at 1, 3, 8, and 24 hours (day 2 before discharge from the clinic). Subjects were scheduled to return to the phase 1 clinic for assessments on days 3, 4, 6, 8, 10, 12, 15, 22, 29, 43, and 57, and at the end-of-study visit on day 85.

**Table 1:** Schedule of events for ZK-001 protocol

Event	Screening (within 35 d of baseline)	Baseline (day –1; within 24 h of day 1)	Post-study treatment administration visits*													
			Day 1 (dosing day)	Day 2 (discharge day)	Day 3	Day 4	Day 6	Day 8	Day 10	Day 12	Day 15	Day 22	Day 29	Day 43	Day 57	Day 85 or early withdrawal
Informed consent	X															
Eligibility	X	X														
Medical history	X	X^†^														
Complete physical examination	X^‡^	X^‡^														X
Study treatment administration			X													
Vital signs^§^	X	X	X^‖^	X^¶^	X	X	X	X	X	X	X	X	X	X	X	X
EKG	X															
Hematology	X	X		X^¶^	X	X	X	X	X	X	X	X	X	X	X	X
Blood chemistry	X	X		X^¶^	X	X	X	X	X	X	X	X	X	X	X	X
Urinalysis	X															
Drug (urine) test	X	X														
Alcohol (breath) test	X	X														
Pregnancy test	X^#^	X^#^														X^#^
Viral markers	X**	X^††^														X^‡‡^
PK sample collection			X^§§^	X^¶^	X	X	X	X	X	X	X	X	X	X	X	X
Adverse events			X^§§^	X^¶^	X	X	X	X	X	X	X	X	X	X	X	X
Concomitant medications	X	X	X^§§^	X^¶^	X	X	X	X	X	X	X	X	X	X	X	X

EKG = electrocardiogram; PK = pharmacokinetics.

* Day 1 included 1-hour (±5 minutes), 3-hour (±30 minutes), and 8-hour (±1 hour) time points post-dosing. Day 2 was 24 hours (±3 hours) post-dosing. Day 3 was 48 hours (±3 hours) post-dosing. Day 4 was 72 hours (±3 hours) post-dosing. Day 6 was 120 hours (±6 hours) post-dosing. Day 8 was 168 hours (±6 hours) post-dosing. Day 10 was 216 hours (±12 hours) post-dosing. Day 12 was 264 hours (±12 hours) post-dosing. Day 15 was 336 hours (±12 hours) post-dosing. Day 22 was 504 hours (±24 hours) post-dosing. Day 29 was 672 hours (±24 hours) post-dosing. Day 43 was 1,008 hours (±48 hours) post-dosing. Day 57 was 1,344 hours (±48 hours) post-dosing. Day 85 was 2,016 hours (±72 hours).

† Update of medical history (as necessary).

‡ Included assessment of body mass index. Height and body weight measured at screening; body weight only measured again at baseline.

§ Vital signs included temperature, sitting blood pressure, respiratory rate, pulse oximetry, and pulse.

‖ Vital signs performed 2 hours (±15 min) and 1 hour (±15 min) prior to dosing, during the IV infusion at 15 min (±5 min) and at the end of the IV infusion (+5 min)], and post-dosing at 1 hour (±5 min), 3 hours (±30 min) and 8 hours (±1 hr).

¶ Performed 24 hours (±3 hours) post-dosing.

# At screening, serum pregnancy test for female subjects of child-bearing potential, and follicle-stimulating hormone assessment for post-menopausal female subjects. The serum pregnancy test was required only for women of childbearing potential at baseline (day –1) and day 85.

** Serology testing for HIV, hepatitis B virus, hepatitis C virus, Dengue virus, West Nile virus, Zika virus (ZIKV) nucleic acid test (NAT) (serum, urine), and ZIKV serology.

†† ZIKV NAT (serum, urine) and ZIKV serology testing.

‡‡ Serology testing for HIV, hepatitis B virus, hepatitis C virus, ZIKV NAT (serum, urine), and ZIKV serology.

§§ PK sample collected, adverse events and concomitant medications assessments at 1 hour (±5 minutes), 3 hours (±30 minutes), and 8 hours (±1 hour) post-dosing. Pre-dose (i.e., baseline) PK sample collected within 2 hours prior to dosing.

### Safety assessment.

The primary outcome was safety, which was a descriptive comparison of incidence and severity (intensity) of any AE among groups of subjects administered ZIKV-Ig versus subjects administered a placebo control for the safety population. The safety of the study drug was assessed by monitoring AEs; laboratory results for blood chemistry, hematology, vital signs, and concomitant medications collected throughout the study; as well as physical examination performed at the end-of-study visit on day 85. AEs were reported spontaneously by the subjects and/or elicited by the PI (or designee) by asking the subjects non-leading questions. All AEs were examined by the PI (or sub-investigator) for assessment of intensity as mild (awareness of a sign or symptom but subject can tolerate), moderate (discomfort enough to cause interference with normal daily activity), or severe (resulting in an inability to do work or do usual daily activity). The association of the AE with the study drug was assessed by the PI as “related” or “not related/no relationship to” the study drug according to International Council for Harmonisation E2A and 21.CFR.312. All AEs were classified according to the *Medical Dictionary for Regulatory Activities* (v. 21.0). In addition, product class-specific AEs such as hypersensitivity (allergic reaction, anaphylaxis), acute renal dysfunction/failure, aseptic meningitis syndrome, hemolysis or hemolytic anemia, thrombotic events, or transfusion-related acute lung injury were of particular interest in this study and were considered AEs of special interest (AESI).

### PK assessment.

The secondary outcomes measure for this study were the PK parameters of ZIKV-Ig. A validated MAGPIX^®^ assay targeting anti-ZIKV antibodies that bind recombinant ZIKV E-protein was used for the primary PK analysis. The MAGPIX (xPONENT software v 4.2, Luminex Corporation, Austin, TX) binding assay is a high-throughput method to quantify antibodies (IgG) binding to ZIKV E-protein. ZIKV E-protein antigen was bound to magnetic carboxylated beads. E-protein binding antibodies present in the diluted clinical serum samples bound to the coupled beads. A phycoerythrin conjugated goat anti-human IgG fragment crystallizable region (Fc) antibody was added to the bound sample. The MAGPIX^®^ instrument detected different distinct wavelengths emitted from the beads, and used them to classify the bead type and determine the median fluorescent intensity of the phycoerythrin-labeled antibody, which was proportional to the amount of bound E-protein antibodies. A seven-point calibration curve was run in each assay using the ZIKV-Ig reference standard, and sample concentrations were calculated from a five-parameter logistic standard curve generated from the calibrator concentrations.

In addition, an xCelligence functional assay (xCelligence System (RTCA software v 2.1.0 Agilent, Santa Clara, CA)) was used to quantify ZIKV neutralization activity of clinical serum samples used in support with the MAGPIX binding PK assay. VeroE6 cells were plated onto 96-well E-plates at a density of 15,000 cells/well, and were then incubated overnight to form a confluent monolayer. Heat-inactivated serum samples were diluted appropriately and incubated with a viral load equivalent to a 100-tissue culture infectious dose in which 50% of cells are infected with ZIKV (strain PRVABC59). Sample and virus mixtures were added to the cell plate then incubated to allow for non-neutralized virus attachment to cells. Next, the sample and virus mixtures were washed from the cells and replaced with serum-free media containing 1% bovine serum albumin to allow cells to remain viable throughout a 3-day incubation period. This incubation period allowed replication of the virus to provide a suitable level on virus-induced cytopathogenicity. Sample end point concentration was determined by interpolation of the signal against a standard curve with known levels of the ZIKV-Ig reference standard.

### Statistical analysis.

The sample size was consistent with a phase 1 first-in-human study and there was no formal sample size calculation. The safety analysis included the safety population that consisted of all subjects who received any amount of study treatment, and was based on the treatment received (ZIKV-Ig or placebo). Descriptive statistics and changes from baseline in continuous clinical laboratory results and vital sign results, as well as frequency of clinically significant findings for clinical laboratory results, vital sign results, and physical examinations are presented by treatment and time point for each test. The PK population included all subjects who received ZIKV-Ig with an adequate number of PK samples (a suitable pre-dose sample and at least one measurable post-dose sample).

The PK parameters included area under the concentration–time curve (AUC) from time 0 to the last quantifiable concentration (AUC_0-t_), AUC from time 0 to day 7 (AUC_0–day 7_), AUC_0-t_ plus the additional area extrapolated to infinity (AUC_0-∞_), the extrapolated area as a percentage of AUC_0-∞_ (AUC_% extrapolated_), the maximum observed concentration (C_max_), the time at which C_max_ occurs (T_max_), the terminal elimination rate constant (λ_z_), the apparent first-order terminal elimination half-life (t_1/2_), total body clearance following IV administration (CL), the volume of distribution following IV administration (V_z_), the adjusted *r*^2^ value for λ_z_ regression, the number of points used in the λ_z_ calculation, and the lower and upper range of times for points used in the λ_z_ calculation.

Serum concentration versus time data were analyzed by standard non-compartmental methods (i.e., linear trapezoidal method for consecutive time points with level or increasing concentrations and log-linear trapezoidal method for consecutive time points with decreasing concentrations with no interpolation). The terminal elimination rate constant, used to determine t_1/2_, was calculated as the negative of the slope of the terminal portion of the observed serum concentration–time curve. In the non-compartmental analysis, concentrations of total antibody less than the lower limit of quantification were set to zero. Actual times and non-nominal times were used in the analysis. The PK parameters were calculated using non-compartmental analysis.

## RESULTS

**Figure 2.**
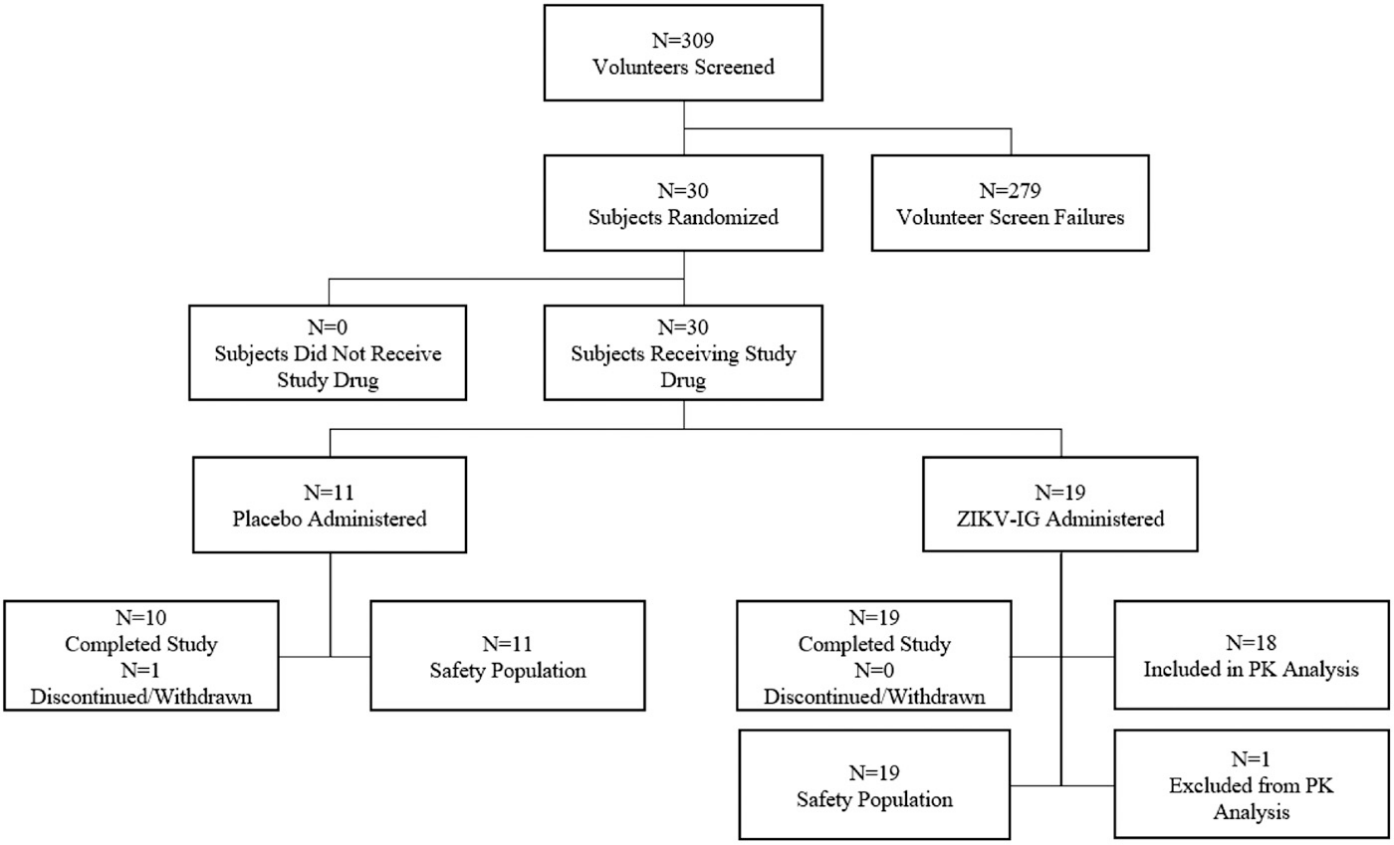
Disposition of volunteers. A total of 309 volunteers were screened for the ZK-001 study. Of these, 279 were screen failures. Thirty subjects met the study eligibility criteria, were randomized, and were administered either human anti-Zika virus immunoglobulin (ZIKV-Ig) (*n* = 19) or placebo (*n* = 11). All 19 subjects in the ZIKV-Ig cohort completed the study, whereas 1 of 11 subjects in the placebo cohort was discontinued from the study (lost to follow-up). All 30 subjects were included in the safety population, and 18 out of 19 subjects who received ZIKV-Ig were included in the pharmacokinetics (PK) population.

The study was conducted from June 27, 2018 to March 6, 2019. A total of 309 volunteers were screened for this study, of which 30 subjects were randomized and assigned sequentially to receive either ZIKV-Ig (*n* = 19) or placebo (*n* = 11) as illustrated in Figure 2. Excluded volunteers (*n* = 279) did not meet inclusion criteria or met exclusion criteria (screen failures), declined to participate (withdrew consent), or met screening criteria but were not enrolled (*n* = 21) (i.e., considered backup subjects).

In total, all 30 randomized subjects received their intended study drug and were included in the safety population. A total of 19 subjects received ZIKV-Ig and 11 subjects received placebo. Each subject received the total planned single-dose volume of 50.0 mL, except one subject in the placebo group who received 49.9978 mL (99.9956%) of the intended 50.0 mL dose. All subjects completed the infusion of the study drug with a mean duration of infusion of 32.7 minutes (range, 32–48 minutes).

All 19 subjects in the ZIKV-Ig cohort completed the study, whereas 1 of 11 subjects (3.3%) in the placebo cohort was discontinued from the study (lost to follow-up). All 30 subjects were included in the safety population, and 18 of 19 subjects who received ZIKV-Ig were included in the PK population. One subject was removed from PK analyses as an outlier. This subject had a much later and lower C_max_ value, with an apparent absorption phase present. It was hypothesized the IV infiltration may have affected the C_max_. For this subject, the infusion started at a 1-mL/min initial infusion rate that was increased to 2 mL/min after 15 minutes (per protocol). Two minutes after the infusion rate increase, the infusion stopped because of the syringe pump high-pressure alarm. The infusion was restarted at a reduced infusion rate of 1 mL/min. Although this subject ultimately received the total 50.0-mL dose, the total infusion duration was 48 minutes. The PI noted in the source notes that it was possible that some of the infusion volume had gone interstitial. There was a non-serious AE reported as swelling in the right antecubital arm (preferred term, peripheral swelling) with mild intensity and not-related causality for this subject. The AE was monitored and was considered resolved the next day.

Of the 30 subjects enrolled, 22 (73.3%) were men and 8 (26.7%) were women, with a mean age of 35.84 years and a baseline body mass index of 24.5 kg/m^2^. The majority of subjects were white (73.3%), and all subjects had blood type O (86.7% had type O+ and 13.3% had type O–). Subject demographics and baseline characteristics are listed in Table 2.

**Table 2:** Subject demographics and baseline characteristics

Parameter	ZIKV-Ig (*n* = 19)	Placebo (*n* = 11)	Total (*N* = 30)
Gender at birth, n (%)			
Male	15 (78.9)	7 (63.6)	22 (73.3)
Female	4 (21.1)	4 (36.4)	8 (26.7)
Childbearing potential, yes	4 (21.1)	4 (36.4)	8 (26.7)
Age, y			
Mean (SD)	35.06 (9.62)	37.18 (13.72)	35.84 (11.12)
Median	35.0	30.0	33.5
Min, max	21.0, 52.0	23.0, 55.0	21.0, 55.0
Race, n (%)			
Asian	3 (15.8)	2 (18.2)	5 (16.7)
Black or African American	1 (5.3)	0 (0)	1 (3.3)
Other	1 (5.3)	1 (9.1)	2 (6.7)
White	14 (73.7)	8 (72.7)	22 (73.3)
Ethnicity, n (%)			
Hispanic or Latino	1 (5.3)	3 (27.3)	4 (13.3)
Not Hispanic or Latino	18 (94.7)	8 (72.7)	26 (86.7)
Height (m)			
Mean (SD)	174.22 (7.86)	172.54 (9.08)	173.60 (8.20)
Median	175.2	170.0	174.3
Min, max	160.1, 186.5	156.5, 186.8	156.5, 186.8
Baseline weight (kg)			
Mean (SD)	75.52 (11.78)	72.10 (14.26)	74.26 (12.60)
Median	75.0	71.0	74.0
Min, max	52.0, 102.0	54.0, 98.0	52.0, 102.0
Baseline body mass index (kg/m^2^)			
Mean (SD)	24.74 (2.38)	24.10 (3.42)	24.50 (2.76)
Median	24.1	23.8	24.1
Min, max	19.9, 30.0	17.6, 29.9	17.6, 30.0
Blood type, n (%)			
O+	17 (89.5)	9 (81.8)	26 (86.7)
O–	2 (10.5)	2 (18.2)	4 (13.3)

Min = minimum; max = maximum, ZIKV-Ig = human anti-Zika virus immunoglobulin.

Twenty of 30 subjects (66.7%) reported 55 AEs. Of these, 12 subjects (63.2%) were from the ZIKV-Ig group compared with eight subjects (72.7%) from the placebo group. There were 51 AEs (92.7%) considered mild in intensity. Four of the 55 AEs (7.3%) in 4 of 30 subjects (13.3%) were considered moderate in intensity, including two AEs in 2 of 19 subjects (10.5%) in the ZIKV-Ig group (lower abdominal pain, streptococcal pharyngitis) and two AEs in 2 of 11 subjects (18.2%) in the placebo group (abdominal abscess, back pain). None of these AEs were considered related to the study drug.

No deaths and no serious adverse events or severe AEs were observed during the study period in either the ZIKV-Ig or placebo study groups. No subject had drug discontinuation (e.g., infusion interruption with a decision not to restart and complete the infusion) as a result of AEs. One subject from the placebo group experienced a not-related AESI of rash reported 2 months after infusion. The mean and median changes from baseline for all hematology and blood chemistry parameters were minimal and similar between the ZIKV-Ig group and the placebo group. There were no related AEs resulting from abnormal vital signs or laboratory values, and no anomalous results were found. There were no clinically significant abnormal vital sign measurements in either treatment group during the study; however, an aspartate aminotransferase elevation (113 U/L) at day 10 in one subject in the ZIKV-Ig group was deemed clinically important by the PI. The PI assessed one subject in the ZIKV-Ig group as having a tender and enlarged submandibular lymph node at end-of-study day 85, indicating it was clinically significant during a physical examination.

The most frequent AEs (> 5% of study subjects) reported included the following: six headache events in five subjects (16.7%), three events of dizziness in three subjects (10.0%), and three events of nausea in two subjects (6.7%). There were two events of fatigue, peripheral swelling, and nasal congestion in two subjects (6.7%) each. There were also two events of increase aspartate aminotransferase and increased blood lactate dehydrogenase in two subjects (6.7%) each. All the remaining reported AEs were reported once in one subjects (3.3%) each, including dysgeusia, paresthesia, abdominal distension, lower abdominal pain, diarrhea, dry mouth, salivary hypersecretion, tongue pigmentation, catheter site hypoesthesia, feeling of body temperature change, influenza-like illness, vessel puncture site bruise, increased alanine aminotransferase, increased blood creatine phosphokinase, increased myoglobin blood, abdominal abscess, hordeolum, oral herpes, streptococcal pharyngitis, upper respiratory tract infection, viral upper respiratory tract infection, oropharyngeal pain, back pain, myalgia, menorrhagia, irregular menstruation, rash, skin lesion, lymphadenopathy, blurred vision, arthropod bite, hypervigilance, and hematuria.

Overall, the most reported related AEs (> 5%) in the safety population (ZIKV-Ig and placebo) were headache (three subjects, 10%) and dizziness (two subjects, 6.7%). Other related AEs reported in one subject (3.3%) each are listed in Table 3. A total of 12 AEs in eight subjects (42.1%) were reported as related to ZIKV-Ig compared with five AEs in three subjects (27.3%) considered as related to placebo. The most frequently reported related AE was headache in three (15.8%) of the ZIKV-Ig-treated subjects; whereas no placebo subjects had treatment-related headache reported. In all three of these subjects, the headache was mild and resolved without treatment and with no sequelae. One subject in the ZIKV-Ig group had 4 of the 12 (33.3%) AEs that the PI considered to be to be related to the drug, including dry mouth, metallic taste in mouth (dysgeusia), paresthesia (left arm), and diarrhea, all of which were mild and resolved without treatment and with no sequelae within 1 to 2 days of onset. Another subject had transient self-resolving mild nausea during the ZIKV-Ig infusion, as well as myalgia (muscle pain) with onset occurring 5 days after ZIKV-Ig infusion that resolved within 3 days after treatment with one dose of 600 mg oral Ibuprofen.

**Table 3:** Adverse events assessed as related to study drug by the *Medical Dictionary for Regulatory Activities *system organ class and preferred term (safety population)

System organ class preferred term	ZIKV-Ig (*n* = 19); m, n (%*)	Placebo (*n* = 11); m, n (%*)	Total (*N* = 30); m, n (%*)
No. of related adverse events and subjects with related adverse events	12, 8 (42.1)	5, 3 (27.3)	17, 11 (36.7)
Nervous system disorders	6, 5 (26.3)	1, 1 (9.1)	7, 6 (20)
Headache	3, 3 (15.8)	0, 0 (0)	3, 3 (10)
Dizziness	1, 1 (5.3)	1, 1 (9.1)	2, 2 (6.7)
Dysgeusia	1, 1 (5.3)	0, 0 (0)	1, 1 (3.3)
Paresthesia	1, 1 (5.3)	0, 0 (0)	1, 1 (3.3)
Gastrointestinal disorders	3, 2 (10.5)	1, 1 (9.1)	4, 3 (10)
Diarrhea	1, 1 (5.3)	0, 0 (0)	1, 1 (3.3)
Dry mouth	1, 1 (5.3)	0, 0 (0)	1, 1 (3.3)
Nausea	1, 1 (5.3)	0, 0 (0)	1, 1 (3.3)
Salivary hypersecretion	0, 0 (0)	1, 1 (9.1)	1, 1 (3.3)
General disorders and administration site conditions	1, 1 (5.3)	2, 2 (18.2)	3, 3 (10)
Catheter site hypoesthesia	1, 1 (5.3)	0, 0 (0)	1, 1 (3.3)
Fatigue	0, 0 (0)	1, 1 (9.1)	1, 1 (3.3)
Feeling of body temperature change	0, 0 (0)	1, 1 (9.1)	1, 1 (3.3)
Musculoskeletal and connective tissue disorders	1, 1 (5.3)	0, 0 (0)	1, 1 (3.3)
Myalgia	1, 1 (5.3)	0, 0 (0)	1, 1 (3.3)
Psychiatric disorders	0, 0 (0)	1, 1 (9.1)	1, 1 (3.3)
Hypervigilance	0, 0 (0)	1, 1 (9.1)	1, 1 (3.3)
Skin and subcutaneous tissue disorders	1, 1 (5.3)	0, 0 (0)	1, 1 (3.3)
Skin lesion	1, 1 (5.3)	0, 0 (0)	1, 1 (3.3)

m = number of adverse events; n = number of subjects with adverse events (incidence); *n *= number of subjects in the analysis population; ZIKV-Ig = human anti-Zika virus immunoglobulin.

* (n/*n*) × 100. Subjects with more than one event in a category are counted once in each category.

**Figure 3.**
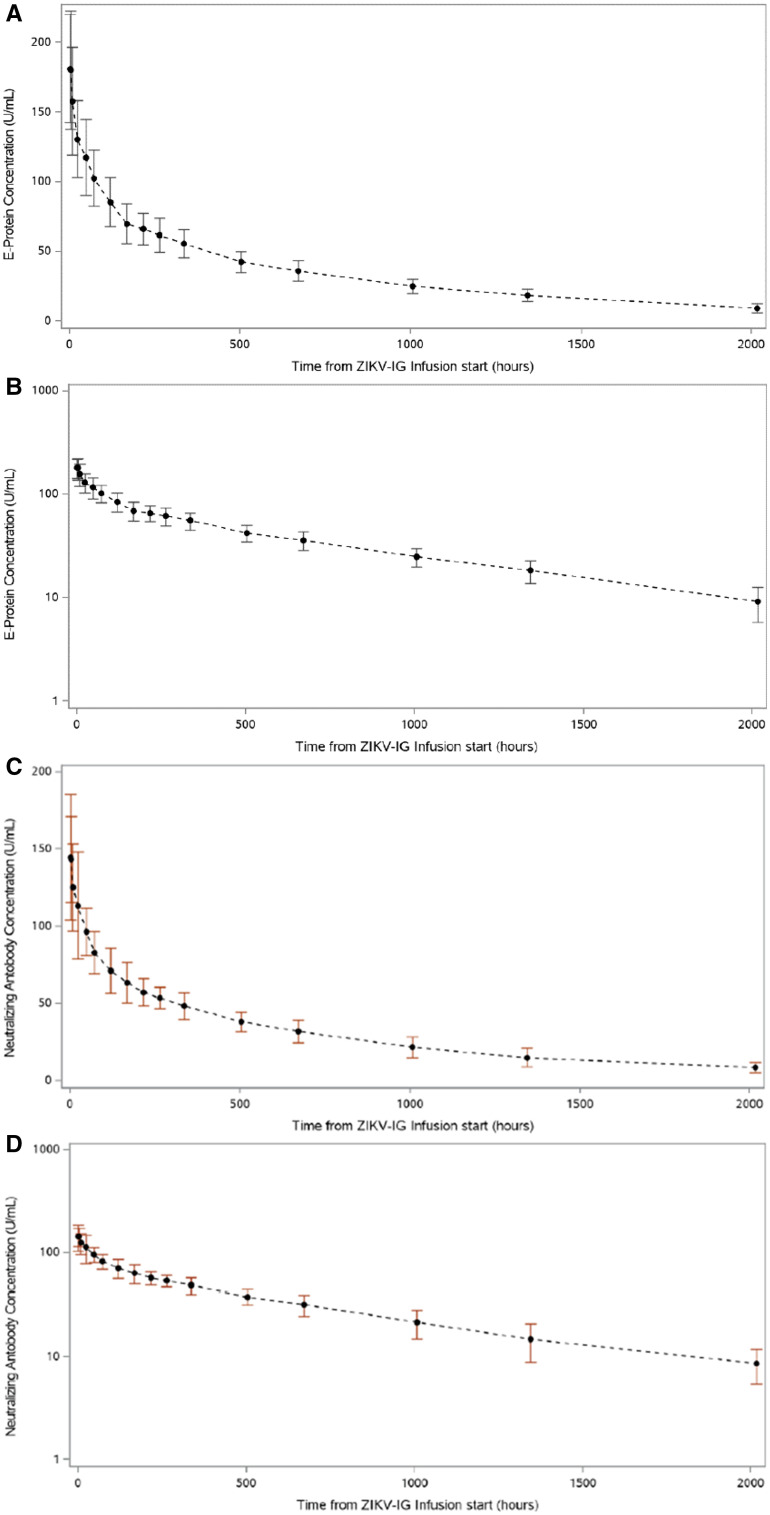
Mean (±SD) concentration–time profiles for anti-Zika virus (ZIKV) E-protein binding antibodies on a linear scale (panel **A**) and a semi-logarithmic scale (panel **B**) [pharmacokinetic (PK) population). Mean (±SD) concentration–time profiles for anti-ZIKV neutralizing antibodies on a linear scale (panel **C**) and a semi-logarithmic scales (panel **D**) (PK population). This figure appears in color at www.ajtmh.org.

PK data were available from pre-dose (i.e., baseline), collected within 2 hours prior to dosing, to end-of-study day 85 for all 19 evaluable subjects who received ZIKV-Ig. After IV administration of ZIKV-Ig, the change in serum concentration of anti-ZIKV E-protein binding antibodies and neutralizing antibodies over time both followed a biphasic disposition, with an initial rapid distribution phase followed by a slow elimination phase (Figure 3).

The summary of ZIKV-Ig PK characteristics of anti-ZIKV E-protein binding antibodies in subjects receiving a single dose, and the PK parameters of ZIKV-Ig based on serum concentrations of ZIKV neutralizing antibodies over time are summarized in Table 4.

**Table 4:** Pharmacokinetic characteristics of Zika virus E-protein binding and neutralizing antibodies (pharmacokinetics population)

PK parameter	Unit	Statistic	E-protein binding antibodies ZIKV-Ig (*n* = 181*)	Neutralizing antibodies ZIKV-Ig (*n* = 181*)
AUC_0–t_	h × U/mL	Geometric mean	67,221	50,235.64
		Geometric CV%	18.8	21.0
		Mean (SD)	68,319 (12,596)	51,299.88 (11,281.39)
		Median	67,700	49,000.2
		Min; max	46,431; 94,737	34,583.0; 83,312.6
AUC_0–7_	h × U/mL	Geometric mean	14,986	12,456.08
		Geometric CV%	25.8	23.6
		Mean (SD)	15,466 (4,198.6)	12,773.22 (2,912.79)
		Median	14,706	12,235.1
		Min; max	9,629; 27,184	8,227.5; 17,803.6
AUC_0–∞_	h × U/mL	Geometric mean	77,224	67,954.72
		Geometric CV%	17.9	23.5
		Mean (SD)	78,374 (13,832)	69,732.01 (16,704.13)
		Median	77,798	66,741.2
		Min; max	55,892; 103,130	45,422.4; 107,532.9
AUC_% extrapolated_	%	Geometric mean	12.27	24.82
		Geometric CV%	33.04	25.0
		Mean (SD)	12.87 (3.99)	25.63 (7.64)
		Median	13.1	23.6
		Min; max	7.1; 19.3	18.3; 50.3
C_max_	U/mL	Geometric mean	182.3	150.15
		Geometric CV%	21.3	26.3
		Mean (SD)	186.3 (41.4)	155.02 (41.26)
		Median	183	146.0
		Min; max	128; 303	95.6; 256.9
t_max_	h	Mean (SD)	2.3 (1.0)	3.83 (5.26)
		Median	2	3.5
		Min; max	2; 4	1.6; 24.5
λ_z_	/h	Geometric mean	0.00103	0.00111
		Geometric CV%	16.38765	43.4
		Mean (SD)	0.00104 (0.00017)	0.00120 (0.00045)
		Median	0.0010	0.0012
		Min; max	0.0008; 0.0014	0.0004; 0.0023
*r*^2^ Value	Proportion	Geometric mean	0.99495	0.94680
		Geometric CV%	1.02524	5.8
		Mean (SD)	0.99500 (0.01004)	0.94823 (0.05203)
		Median	0.9983	0.9545
		Min; max	0.9577; 1.0000	0.7923; 0.9976
No. of points used in λ_z_ calculation	Count	Geometric mean	4.1	5.70
		Geometric CV%	45.5	52.2
		Mean (SD)	4.6 (2.4)	6.39 (3.18)
		Median	4	5.5
		Min; max	3; 10	3.0; 13.0
t_1/2_	h	Geometric mean	674.7	624.76
		Geometric CV%	16.4	43.4
		Mean (SD)	683.1 (110.8)	683.34 (340.23)
		Median	686	586.6
		Min; max	508; 889	304.2; 1,772.7
CL	mL/h	Geometric mean	6.871	7.808
		Geometric CV%	17.9	23.5
		Mean (SD)	7.0 (1.3)	8.01 (1.84)
		Median	7	8.0
		Min; max	5; 9	4.9; 11.7
V_z_	mL	Geometric mean	6,687.7	7,037.73
		Geometric CV%	20.1	26.4
		Mean (SD)	6,809.9 (1,299.2)	7,286.78 (2,181.80)
		Median	6,865	6,570.1
		Min; max	4,318; 9,426	5,126.3; 13,141.3
λ_z_ lower limit	h	Mean (SD)	705.7 (337.4)	324.78 (243.12)
		Median	674	276.2
		Min; max	118; 1,055	8.6; 672.9
λ_z_ upper limit	h	Mean (SD)	1,937.8 (216.7)	1,267.20 (355.54)
		Median	2,016	1,344.1
		Min; max	1,345; 2,041	672.4; 2,016.2

AUC = area under the concentration–time curve; CV = coefficient of variation; max = maximum; min = minimum; PK = pharmacokinetics; ZIKV-Ig = human anti-Zika virus immunoglobulin; AUC_0-t_ = area under the concentration-time curve from time 0 to the last quantifiable concentration; AUC_0-day 7_ = area under the concentration-time curve from time 0 to day 7; AUC_0-∞_ = AUC_0-t_ plus the additional area extrapolated to infinity; AUC_% extrapolated_ = extrapolated area as a percentage of AUC_0-∞_; C_max_ = maximum observed concentration; T_max_ = time at which C_max_ occurs; λ_z_ = terminal elimination rate constant; t_1/2_ = apparent first order terminal elimination half-life; CL = total body clearance following IV administration; V_z_ = volume of distribution following IV administration.

* One subject was removed from PK analyses as an outlier.

## DISCUSSION

Currently, there is no approved therapeutic or licensed ZIKV vaccines to prevent ZIKV infection or disease, including in populations at risk, such as pregnant women. Passive immunization with an antibody therapeutic such as ZIKV-Ig could provide protection against ZIKV infection (with regular dosing to maintain ZIKV antibody levels during pregnancy). EBCI is developing ZIKV-Ig, a human hyperimmune product of purified IgG fraction of human plasma containing antibodies to ZIKV as an intervention to ZIKV to address this unmet need. ZIKV-Ig was manufactured using the same hyperimmune manufacturing platform process as the one used to manufacture U.S. FDA–licensed products.

The selection of the ZIKV-Ig dose level of 50.0 mL ZIKV-Ig (∼0.1 g/kg), containing a total of 4.65 g protein, was estimated to yield protective levels of ZIKV antibodies based on immune-correlated data from ZIKV infection studies^18^,^19^ in nonhuman primates (NHPs) and is within the efficacious ZIKV-Ig doses (0.05–0.4 g/kg) evaluated in the mouse model of ZIKV infection.^20^ The selected 50.0-mL dose of ZIKV-Ig falls within the range of doses used for the other products manufactured with EBCI’s hyperimmune platform and is considered a low Ig IV dose because standard Ig IV doses range from 0.2 to 1 g/kg.

Igs are normal constituents of blood, and they are used at physiological levels without creating pharmacological and toxicological active metabolites.^21^ The safety profile for Ig products is well established as a result of a long history of their use in clinical practice for a range of medical conditions.^17^,^18^,^22^ The most common types of AEs related to IV Ig and hyperimmune products are non-anaphylactic infusion reactions (self-limiting), such as abdominal or back pain, fever, headache, chills, rash, fatigue, nausea, or vomiting. The incidence of adverse reactions associated with IV Ig’s is in the range of 1% to 15%, but is typically ≤ 5%. AESI such as hypersensitivity reactions, renal dysfunction/failure, aseptic meningitis syndrome, hemolysis, transfusion-related acute lung injury, and thrombotic events have been reported infrequently with IV Ig. ZIKV-Ig was safe and well tolerated in healthy adult subjects with blood types O+ and O–. Related AEs that were reported more frequently in the ZIKV-Ig group compared with the placebo group included headaches. These were reported only in ZIKV-Ig group, which is an expected type of related AE (i.e., adverse drug reaction) for a human IV Ig product. There were no AESI reported in the 19 subjects administered ZIKV-Ig in our study, suggesting a similar safety profile to other IV Ig products.

The PK parameters were characterized with a validated binding assay (using a recombinant ZIKV E-protein antigen) and were confirmed further by a functional ZIKV neutralization assay. The xCelligence functional neutralization assay results were also similar to the results obtained by the MAGPIX binding assay, although they were slightly lower, as expected, because neutralizing antibodies are a subset of binding antibodies. Overall, both assays used to analyze the PK concentrations in our study demonstrated similar PK trends for ZIKV-Ig. The PK parameters for ZIKV-Ig were similar to PK parameters of other human IV Ig products manufactured using EBCI’s hyperimmune platform and other commercial IV Ig products. Although the doses and protein concentrations vary by products and indication, the drug product is primarily purified human IgG and is expected to have a similar half-life to other commercial products (e.g., (HepaGam B^®^ (Hepatitis B Immune Globulin Intravenous [Human]), Emergent BioSolutions, Winnipeg, Canada, VariZIG^®^ (Varicella Zoster Immune Globulin [Human)]; Emergent BioSolutions, Winnipeg, Canada, VIGIV^®^ Vaccinia Immune Globulin Intravenous (Human); Emergent BioSolutions, Winnipeg, Canada, ANTHRASIL^®^ (Anthrax Immune Globulin Intravenous [Human]; Emergent BioSolutions, Winnipeg, Canada).). As demonstrated in our study, ZIKV-Ig had a calculated mean half-life of 28.1 days, which is in the range of the expected half-life for this product class (i.e., 21–30 days). The C_max_, T_max_, and AUC of ZIKV-Ig were also consistent with those of human-derived IgG products.

In conclusion, ZIKV-Ig appears safe and was well-tolerated in healthy adult subjects with blood types O+ and O–, and had PK parameters consistent with the expected PK of other commercially available human hyperimmune products.

## Supplemental Material


Supplemental materials
