# Supplementary material for: Results of a Double-Blind, Randomized, Placebo-Controlled Phase 1 Study to Evaluate the Safety and Pharmacokinetics of Anti-Zika Virus Immunoglobulin
Source: Am J Trop Med Hyg. 2021 Oct 4;105(6):1552–62. doi: 10.4269/ajtmh.20-1578 (PMC8641324; doi:10.4269/ajtmh.20-1578)
Supplement: Supplementary file 1 [file tpmd201578.SD1.pdf]

Appendix 1

Inclusion Criteria

The inclusion criteria included the following:

1. Informed consent voluntarily signed by subject.

2. Age: 18 to 55 years.

3. Blood type O_+ or O_-.

4. Body mass index (BMI) of 18 to 30 (minimum body weight of 50 kg).

5. For female subjects (with male partners) that were not surgically sterilized (e.g., did not undergo hysterectomy, bilateral oophorectomy or tubal ligation), use of an effective method of contraception throughout the study including:

- Using hormonal contraception (oral, injectable or implant) continuously for three months prior to screening and were willing to continue to use hormonal contraception throughout the entire study.
- Intrauterine device (IUD) placed at least one month prior to screening.
- Double barrier type of birth control measure (e.g., condoms, diaphragms, cervical sponge with spermicide).
- True abstinence.
- For female subjects who were post-menopausal, documented follicle-stimulating hormone (FSH) ≥40 mIU/mL must be obtained. If the FSH is <40 mIU/mL, the subject must agree to use an acceptable form of contraception (see above).
- Females of childbearing potential without male sexual partners must have been willing to maintain their sexual status as it is throughout the study.

6. For male subjects (with female partners) that did not have a vasectomy, use of a condom with spermicide or true abstinence for the duration of the study. Note: female partners (that were of childbearing potential) of male study subjects (that did not have a vasectomy) used one of the effective contraception methods (e.g., hormonal contraception, IUD or barrier type). Also, male subjects could not donate sperm for the duration of the study. Males without female sexual partners were willing to maintain their sexual status as it was throughout the study.

7. Healthy as determined by the Principal Investigator (or a qualified designate) based on medical history, physical exam, vital signs, urinalysis, blood chemistry and hematology test results at screening.

Exclusion Criteria

These exclusion criteria were selected to prevent (or greatly reduce) the enrollment of nonhealthy subjects and/or to prevent the enrollment of subjects for whom PK assays may be negatively affected due to pre-exposure to, or concomitant infection with flavivirus. Volunteers who had any of the following exclusion criteria at screening and/or baseline were rejected from participation in the study:

1. Use of any investigational product within the past 30 days.
2. Use of any investigational product during the study.
3. Individuals with blood type A, B or AB.
4. Recipient of any blood product within the past 12 months.
5. Plasma donation within 7 days or significant blood loss or blood donation within 56 days of baseline.
6. Blood donation at any time during the study.
7. Females with a hemoglobin level ≤120 g/L.

8. Males with a hemoglobin level <130 g/L.

9. History of hypersensitivity to blood or plasma products.

10. History of allergy to latex or rubber.

11. History of IgA deficiency.

12. History of hypercoagulable conditions (e.g., deep vein thrombosis or pulmonary embolism).

13. History of myocardial infarction.

14. History of stroke.

15. History of renal impairment/failure.

16. Currently pregnant or lactating or planning to become pregnant during the study.

17. History of flavivirus infection (*ZIKV*, *Dengue virus*, *West Nile virus*, *Japanese encephalitis virus*, *Yellow fever virus*) or vaccination with licensed or investigational *Flavivirus* vaccine.
Note: Pre-exposure could negatively affect assay results.

18. Plans to travel to an area with active flavivirus (e.g., *ZIKV* and/or DENV) transmission during the study (and up to 10 months after study drug administration) or has returned from an endemic area with these diseases within 30 days of screening.
Note: Subjects were also advised to recommend to their partner(s) (if applicable), not to travel to flavivirus (e.g., *ZIKV* and/or DENV) endemic regions for the duration of the study and up to 10 months after the study drug administration [i.e., from screening until end of the study visit at Day 85, and up to 6 months after last in-clinic visit at Day 85)].

19. Positive nucleic acid test (NAT) or serology for *ZIKV* (at screening) or positive serology for WNV or DENV (at screening). Note: Pre-exposure could negatively affect assay results.

20. Positive serology test (at screening) for human immunodeficiency virus 1 and 2 (HIV), hepatitis C virus (HCV); positive test for hepatitis B virus (HBV) as determined by HBsAg.

21. History of chronic or acute severe neurologic condition (e.g., diagnosis of Guillain-Barre syndrome, epilepsy, Bell's palsy, meningitis or disease with any focal neurologic deficits).
22. Heavy smokers (greater than 15 cigarettes a day) or electronic cigarette use.
23. History of, or suspected substance abuse problem (including alcohol).
24. Failure of drug (urine) test at screening or baseline.
25. Failure of alcohol (breath) test at screening or baseline.
26. Receipt of a live vaccine within 28 days prior to screening or anticipated receipt of a live vaccine during the study period.
27. Individuals with planned surgical procedures that will occur during the study.
28. An opinion of the Investigator that it would be unwise to allow participation of the subject in the study.

Note: with respect to exclusion criterion no. 18, subjects were to recommend to their partner(s) (if applicable) not to travel to flavivirus (e.g., *ZIKV* and/or *DENV*) endemic regions for the duration of the study and up to 10 months after the study drug administration.

Appendix 2
Schedule of Events for ZK-001 Protocol

	Screening (within 35 days of Baseline)	Baseline (Day -1; within 24 hrs of Day 1)	Post Study Treatment Administration Visits*													
			Day 1 (Dosing Day)	Day 2 (Discharge Day)	D3	D4	D6	D8	D10	D12	D15	D22	D29	D43	D57	Day 85 or Early Withdrawal
Informed consent	X															
Eligibility	X	X														
Medical history	X	X[1]														
Complete physical exam	X[2]	X[2]														X
Study treatment administration			X													
Vital signs[3]	X	X	X[4a]	X[5]	X	X	X	X	X	X	X	X	X	X	X	X
ECG	X															
Hematology	X	X		X[5]	X	X	X	X	X	X	X	X	X	X	X	X
Blood chemistry	X	X		X[5]	X	X	X	X	X	X	X	X	X	X	X	X
Urinalysis	X															
Drug (urine) test	X	X														
Alcohol (breath) test	X	X														
Pregnancy test	X[6]	X[6]														X[6]
Viral markers	X[7]	X[8]														X[9]
PK sample collection			X[4b]	X[5]	X	X	X	X	X	X	X	X	X	X	X	X
Adverse events			X[4b]	X[5]	X	X	X	X	X	X	X	X	X	X	X	X
Concomitant medications	X	X	X[4b]	X[5]	X	X	X	X	X	X	X	X	X	X	X	X

[1] Updated of medical history (as was necessary).

[2] Included assessment of BMI; height and body weight will be measured at Screening and only body weight again at Baseline.

[3] Vital signs included temperature, sitting blood pressure, respiratory rate, pulse oximetry and pulse.

[4a] Vital signs performed 2 hours (±15 min) and 1 hour (±15 min) prior to dosing, during the IV infusion at 15 min (±5 min) and at the end of the IV infusion (+5 min)], and post-dosing at 1 hour (±5 min), 3 hours (±30 min) and 8 hours (±1 hr).

[4b] PK sample collected, adverse events and concomitant medications assessments at 1 hour (±5 min), 3 hours (±30 min) and 8 hours (±1 hr) post-dosing. Pre-dose (i.e., baseline) PK sample collected within 2 hours prior to dosing.

[5] Performed 24 hours (±3 hours) post-dosing.

[6] At screening serum pregnancy test for female subjects of child-bearing potential and FSH assessment for post-menopausal female subjects. The serum pregnancy test was required only for women of childbearing potential for Baseline (Day -1) and Day 85 study visits.

[7] Serology testing for HIV, HBV, HCV, *DENV*, *WNV*; *ZIKV* NAT (serum, urine) and *ZIKV* serology.

[8] *ZIKV* NAT (serum, urine) and *ZIKV* serology testing.

[9] Serology testing for HIV, HBV, HCV; *ZIKV* NAT (serum, urine) and *ZIKV* serology.

* Day 1 included 1 hour (±5 min), 3 hours (±30 min) and 8 hours (±1 hr) time-points post-dosing, Day 2 is 24 hours (±3 hrs), Day 3 is 48 hours (±3 hrs), Day 4 is 72 hours (±3 hrs), Day 6 is 120 hours (±6 hrs), Day 8 is 168 hours (±6 hrs), Day 10 is 216 hours (±12 hrs), Day 12 is 264 hours (±12 hrs), Day 15 is 336 hours (±12 hrs), Day 22 is 504 hours (±24 hrs), Day 29 is 672 hours (±24 hrs), Day 43 is 1008 hours (±48 hrs), Day 57 is 1344 hours (±48 hrs) and Day 85 is 2016 hours (±72 hrs) post-dosing.
